# Supplementary material for: Novel Concept of Alpha Satellite Cascading Higher-Order Repeats (HORs) and Precise Identification of 15mer and 20mer Cascading HORs in Complete T2T-CHM13 Assembly of Human Chromosome 15
Source: Int J Mol Sci. 2024 Apr 16;25(8):4395. doi: 10.3390/ijms25084395 (PMC11050224; doi:10.3390/ijms25084395)
Supplement: Supplementary file 1 [file ijms-25-04395-s001.zip › supplementary_materials.pdf]

Supplementary Materials for

Novel concept of alpha satellite Cascading Higher Order Repeats (HORs) and exemplification of active 15mer Cascading HOR in T2T-CHM13 assembly of human chromosome 15

Matko Glunčić, Ines Vlahović, Marija Rosandić, Vladimir Paar

Corresponding author: matko@phy.hr

The Supplementary file includes:

Figs. S1 to S4
Tables S1 to S5

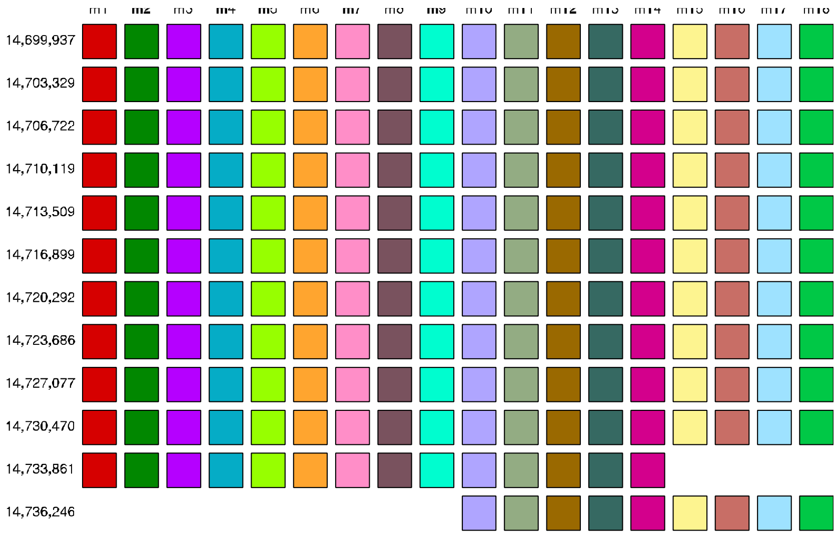

Figure S1. Willard's type 18mer alpha satellite HOR alignment (hor01). Start position 14,699,937 bp and end position 14,737,927 bp in T2T-CHM13. The numbers on the left side indicate the initial position of the first monomer in each HOR copy.

Figure S2. (separate file) Willard's type 25mer/26mer alpha alpha satellite HOR alignment (hor02). Start position 15,417,939 bp and end position 15,692,443 bp in T2T-CHM 13. The numbers on the left side indicate the initial position of the first monomer in each HOR copy.

Figure S3. (separate file) Cascading 15mer alpha satellite HOR alignment (hor03). Start position 16679039 bp and end position 17,683,163 bp in T2T- CHM 13. The numbers on the left side indicate the initial position of the first monomer in each HOR and SubHOR copy.

Figure S4. (separate file) Cascading 20mer alpha satellite HOR alignment (hor04). Start position 15,993,645 bp and end position 16,555,446 bp in T2T- CHM 13. The numbers on the left side indicate the initial position of the first monomer in each HOR and SubHOR copy.

Table S1. Number of 15mer variant HOR copies in hor03 array. Labels for individual variants of HOR copies are provided in Figure Fig. 3C.

Variant HOR copy	No
a	279
a'	62
a*	4
b	5
b'	1
c	16
c'	21
d	7
d'	13
e	17
f	4
**Total**	**429**

Table S2. Canonical 18mer consensus sequence.

Monomer type t1
TGAGAAACTGTACTGTGATGTGTGCATCCATCTCACAAAGGTGAAACTTCCTTTT
GATTGAGCAGTTTTGAAACACTCTTTTTGTACAATCTGCAAGTGGATATTTGGAG
CCCTTTGGGGCCCATGGTGGAAAAGGAAATATCTTCACATAAAAACTACACAG
AAGCATTC
Monomer type t2
CGAGTAGCTTCTTTGTGATGCGTGCGTTCATCTCACAGAGTTGAGCCTTTCTTTTG
ATTGAGCAGTTTTGAAGCACACTTCTTGTGGAATCTGCAAATGGATATTTGGAG
CGCTTTGCAGCCTATGGGGGAAAAGGAAATATCTTCGTATAAAAGCTAGACAG
AATCACTC
Monomer type t3
TCAGAAACTTCTTTGTGATGTGTACGCTCATCTCACAGAATTCAACCTTGCTTTT
GTTTGAGAAGTTTTGAAACACTCTTTTTTTAGTATCTGCAAGCAGATATTTGGAG
TGGTTTGGGGCCTATGGTGGAAAAGGAAATATCATCACATAAAAGCTCGACAG
AAGCATTC
Monomer type t4
TAAGAAACTTCTTTCTGATAAGTGCATTCATCTCACAGAGTTCAGCCTTTCTTTTG
ATTGAGCAGCTGTGAAACACTCTTTTTGTAGAATCTGCAAGTGTATATTTGGAGT
GCTTTGCGGCCAATGGTGGAAAAGGAAATACCTTCACATAAAAACTAGACAGA
AGCATTC
Monomer type t5
TGAGATACAGCTTTGTGATGTGTGCATTCGTCTCATAGTGTTAAACCTTTCTTTTG
ATGGTTCAGTTTTGAAACACTCTTTTTGTAGAATCTGGAAGTGGATATTTGGAGC
GCTTTGAGGCCTATGATGGAAAAGGAAATGTCTTCACTTAAAAACGAGACAGA
AGCATTC
Monomer type t6
TGAGAGACTTCTCTGTGAGGTGTCCATTCATCTCGCAGAGTTGAACCTTTCTTTT
CCTTGAGCAGTTTTGAAACCCTCTGTAGAATCTGCAAGTGGAGATTTAGAGTTCT
TTGAGTCCTATGGTGGAAAAGGAAATATCTTCACATAAAAACTAGACAGAAGC
ATTC
Monomer type t7
TGAGAAACCTCTTTGTGATTTGTGCATTCATCTCACAGAGTTGAACCTTTCTTTCA
ATTGAACAGTTTTGAAACAGTCTTTTTGTGGAATCTGCAATTGGATATATGGAGC
GCTTTGAGGCCTATGGTTGAAAAGCATTATCTTCACATAAAAACATCACAGAAG
AATTC
Monomer type t8
TGAGAAACTACTTTGTGATGTATACATTCATCTCACAGAGTTGAATGAGCAGTTT
CTTTTGATTGAGCAGTTTTGAAAAACTCTTTATGTAGAATCTGCAAGTGTGTATT

TGGAGCACTTTGCGGCCTATGTGGGAAAAGGAATTATCTTCGCACAAAAACTAG
GCAGAAACATTC
Monomer type t9
TGAGAAACTACTTTGTGATGTGTGCATGCATCTCACAGAGTTGAGTCTTTCTTTT
GATTGAGCAGTTTTGAAACAGTCTCTTTGTAGAAACTGCCAGTAGATATTTGGG
ACGCCTTGTGGCCTATGGTGAAAAGGGAAATATCTTCACACAAAAACGGAAGC
ATTC
Monomer type t10
TGGGAAACTTCTTTTTGATGTGTGCATTCATCTCACAGAGTTGAACCTGTCTTTTT
ATTGAGCAGTTTTGAAACACTCTTTTTGTAGAATCTGCAAGTGAATATTTGGAGC
ACTTTGAGGCCTATGGTGGAAAAGGAAATATCTTCACATAAAAACTAGACAGA
AGCACTC
Monomer type t11
TGTGAAACTTCCTTGTAATGTATGCGTCCATCACACAAAGTTGAACCTTTGTTTT
AATTGAGTAGTTAAGAAACACCTTTTCGGAGAATCTGCAAGTGGATATTTGGCA
TGATTTGCGGCCTATGGTGTAAAAGGCAATACCTTCATATAAAAACTAGACAGA
AGTATTC
Monomer type t12
TGAGAAACTCCTTTGTGATGTGTGCATTCATCTCACAGTGTTTAACCTATCTTTTG
ACTGAGCAGTTTGGAAACCCTCTTTTGGAGAATGTGTAAGTGGATATTTGAAGG
TTTTTGAGGACTATGGTGGAAAAGGAAATACCTTCACCTAAAATCTAGGCAGAA
GCATTC
Monomer type t13
TGAGAAACTTCTTTGTGATGTGTGCATTCATCACATAAAGGTGAAACTTTCTTTT
GATTGAGCAGTTTGCAACTCTGCTTTTCTAGAGTCTGCTATTGGATATTTGGAGC
GATTTGCAGCCTATGGTGTAAAAGAAAATATCTTCACATAAAAAGTAGACAGA
AGCATTC
Monomer type t14
TGAGAAGCTTCTTTGTGATGTGTGCGTTCATCTCACAGAGTTGTACATTTCTTTTG
ATGGAGCAGTTTTGAAACACTCTCTTTGTAGAACCTGCAAGAGGATATTTGGAG
CGCTTTGTGGTCTATGGTGGAAAAGGCAATATCTTCCCATAAAAACCAGACAGA
AGCATTC
Monomer type t15
TGAGAAACTTCTTTGTGACGGGTGCATTCATCACACAGAGTTGAACCTTTCTTTT
GATTGAACATTTTTGAAAAACTCTTTTTGTATAATCTGCAAGTGGATATTTGGAG
CGCTTTGAGGCCCTTGCTGGAAAAGGAAATATCTTCACATAAAATCTAGACAGA
TCATTC
Monomer type t16
TCCCAAAGCGCTGGGATTACAGGCGTGAGCCACCGCGCCCGGCCTGAACTATTC
TTTTGATTGAGCAGTGTGGAGACAGTCTTTTGGTAGTATCTGAAAATGGATATTA

GGAGCGCTTTGAATCCTATAGAGCAGAAGGAAATATCTTCACATAAAAACTAA
TCAGAAGCATTC
Monomer type t17
TGAGAAGCTGTTTTTTGATGTGTGCATTCACCCCACAGAGTTGAACTTTTCTTTTG
ATTGAGAAGTATTGAAACTCTCTTTTTGTAGAATTTACAAGTGGATACTTGGAGG
GCTTTGAGGCCTACGGTGGAAAAGGAAATATCTTCACATAAAAACTAGACAGA
AGCATTC
Monomer type t18
TGAGAATCTTCTTTGTGAGGTGTGCGTTCATCTCACAGAGTTGAACTTTTTTTTTA
TTGGGCAGTTTTGAAACACTCTTTTTGTGGAATCTGAAAATGGATATTTGGAGTG
CTTTGTGGCCTATGGTGGAACAGGAAATATCTTCATATAAAAACTAGACAGAGG
CATTC

Table S3. Canonical 25mer and 26mer consensus sequence.

Monomer type t1
TGAGATTCTTCTTTGTGAGGTGTGCATTCAACTCACAGAGTTGAACTTATCTTTTC
CTTGAGCACTTTCATATCTCATTTCTGTAGAATCTGCAAGTGGATATTTGGAGC
TCTTTGCACCCTGTGGTGGAAAGGGAACTATCTTCATATAAAAACTACAAAGAA
GCATTC
Monomer type t2
AGAGAAACTTCTTGTGATGAATGCATTCCTCACACAGAGCTGAACCTTTCTTTTT
ATTGAGCAGTATTGAAACGCTCTTTTTGCAGAATCACCAAGTGGATATTTGGAG
AGCTTTGGGGCCTGTTTTGGAAAATGAAATATCTTCAAAGTAAAACTACACAGA
ACCATTC
Monomer type t3
TGAGAAACTTCTTTATGATGTGTGCATTCAACTCTCAGAGTTGAACCTACCTTAT
GATTGAGCAATTTGGAAACACTCTTTTTGTAGAGCCTGCAAGTGGATATTTAGA
ACGATTTGAGGCCTATTGTGGAAAAGCAAATATCTTCACATAAAAACTACACA
GAAGCATTC
Monomer type t4
TGAGAAACTTCTTTGGCATGTGTGCATTCAACTAACAGTGTTGAACGTATCTTTT
GATTGAGCAGCTTAGAATCTCTCTTTTTGTAGAAAATGCAAGTAGATATTTGGA
GCCCCATTTTGCCCTATGGTAGAAAACAAAACATCTTCACATAAAATCTACACA
GAAGCATTC
Monomer type t5
TGAGAAACTTCTTTGTGATGTTTGCATTGAACTCCCAGAGTCGAACCTATCTTTT
GATAGAGCACTTTTGTATCTCTCTTTTTGCGGAATCTGCAAGTGGATATTTGGAA
AGCTTGAGGCCTATTGTGAAAAAGGAAATATCTTCACATAAAAACTACAGAGA
AGCATTC
Monomer type t6
TGAGAAACTTCTTTGTGAGGCATGGATTCAACCCACAGAGTTGGACTTATCATT
GAGCAGTTTTGAATCTCTCTTTTTGTCGAATCTGCAAGTGGATATTTGGAGCCCT
TTGCAACCTAGGGTGGAAAAGGAAATACCTTCAAATAAAAACTATATAGAAGC
ATTCCG
Monomer type t7
TAAAACTTCTTTGTGATGTGTGCATTCGTCTCACAGAGTTGAACCTATCTAATGA
TTGAGCGGTTTTGAAACACTCATTTTGTAGAACCTGCAAGTGGATATTGGGAGT
ACTTTGTGGCCTTCTTTGGAAAAGGGAATATCTTCACATAAAAACTACAAAGAA
GCATTC
Monomer type t8
TGAGAAACTTCTTTGTGATGTGTGCATTCATCTCACAGTGTTGGACGTTTCTTTTG
ATAGGGCAGTTTTGAAACACTCTTTTTCTAGAATCTGCAAGTGGATATTTGGAGC

GCTTTGAGGCCTAATGTGGAAAATCAAATATCTTCACATAAAAACTACACAGA
GGCATTC
Monomer type t9
TGAGAAACTTCTTTTTGTGTGTGCATTCAACTCACATAGTTGAAGTAATCTTTG
GATTTAGCTGTTTTGAATCTCCTTTTTGCAGAATCTGCAAGTTGATACTTGGAGC
CCTGTTTCACCCTATAGTGGAAAAGCAAATATCTTCACATAAACAAACCCTACA
GAGAAGCATTC
Monomer type t10
AGAGAAAGTCCTTTGTGATGTGTGCATTGAACATGCAGAGTTGACACTATCTTTT
GATTGTACAGTTTTGAATACGTCTTTTTGTAGAATCTGCAAGTGGAAGTTTGGAG
CTGTTTGCACCCTGTGGTGTAAAAGGAAATATCTTCATATAAAAGCTACACAGA
AGCAT
Monomer type t11
AGAGAAAGTCCTTTGTGATGTGTGCATTGAACACGCAGAGTTGAAACTATCTTT
TGATTGTACAGTTTTGAATATCTCTTTTTGTAGAATCTGCAAGTGGAAGTTTGGA
GCTGTTTGCACGCTGTGGTGCAAAAGGAAATATCTTCATATAAAAACTACACAG
AAGCTTTC
Monomer type t12
TCAGAAAGACTTCTTTGTGATGAATGCGTTCCTCACACAGAGTTGAATCTTCCTT
TTTATTGAGTAGTATTGAAACCCTCTTTTTGCAGAATAACCAGGTGGATATTTGG
AGAGCTTTGAGGCCTGTTTTGGAAAAGCAAATATCTTCAAATTAAAACCACACA
GAAGCATTC
Monomer type t13
TGAGAAGCTTCTTTGTGATGTGTGCATTCAACTCTCAGAGTTCAACGTGTCTTAT
GATGGAGCAGTTTGGAAACACTCTTTTTTGTAGAAACTGCAAGTGGATATGTAG
AGCGATTTGAGGCCTACTGTGGAAAAGCAAATATCTTCACATAACAACTACAC
AGAAGCACTCC
Monomer type t14
TAGAAACTTCTTTGTGATGTGTGAATTCAACTCACAGAGCTGAACCTATCTTTTG
ATGGAGTAGCTTAGAATCTCTCTTTTTTTAGAATCTGCACGTGGATATTTGGAGC
GCTTTGAGACCTAAAGTGGAAAAGCAAATATCTTCACATAAAATCTACATAGA
GGCACTC
Monomer type t15
TAAGAAACTTCTTTTTGATGTGTGCATTCACCTCACAGAGCTGAACCGATCCTTC
GAGTGACCAGTTTTGAATCTCTCTTTTTATACAATCTGCAAGTGGATATTTGGAG
CCCTTTGCGGCCTATGGTGGAAAAGGAAATATCTTCAAATAAAAACTACACAG
AAGCATTC
Monomer type t16
TAAGAAACTTCTTTTTGATGTGTGCATTCAACTCACAGAGCTGAAGCACACAGT
GCTTGAGTGACCAGTTTTGAATCTCTCTTTTTGTACAATCTGCAAGTGGATATTG

GGAGCCCTTTGCGGCCTGTGGTGGAAAAGGAAATATCTTCAAATAAAAACTAC
ACAGAAGCATTC
Monomer type t17
TGAGAAACTTCTTTGTGATGTGTACATTCATCTCACAGAGTTGACAATTTCTTTTG
ATTGAGCAGTTTTGAAACACTGCTTTTGTAGAGTCTGGAAGTTGATATTTGGAGG
GCTTTGAGGTCTATTTCGGAAAAGAAAATATCTTCACTTAAAAACTAGGCAGAA
ATACTG
Monomer type t18
TGAGAAACTTCTTTGTTATGTGAGCATTCAACTCACAGAGTTGAACCTATCTTTT
GATTGAGCAGTTTTGAATCTCTCATTTTGCAGAATCTGCAAGGGGATATTTGGAG
CCCTTTGCGGCCTATGGTGGAAAAGGAAATACCTTCAAATGAAAAGCACACAG
AGGCATTC
Monomer type t19
TGAGAAACTTCCTCGTGATTGTGCATTCAACTCACAGAGTTAAACCTATCTTATG
ATTGACCAGTTTTGGAACACTCTTTTCATAGGATCTGCAAGTGGATATTTGGCGT
GCTTTGAGGCCTATCGTGGAAAAGCAAATAACTTCAGATAAAAACTATACAGA
AGCATTC
Monomer type t20
TGAGAAACTTCTTTGTGATGTGTGCATTGATCTCACAGAGTTGAAAGTGTATTTT
GATTGAGCAGTTTTGAAACACTCTTTTTGTAGAATCTGCAAGTGGATAATTGGG
GAGATTTGAGGTATATTGTGGAAAAGCAAGTATCTTCATATAAAAACTATACAG
AAGCTTTC
Monomer type t21
TGAGAAACATCTTTGTGAGGTTTGCATTCAACTCACAGAGCTGGAACTATCTTTT
GAGTGACCAGTTTTGAATCTCTCTTTTTGTACAATCTGCAAGTGGATATTTGGAG
CGTTTTGAGGCCTACATTTGAAAATCAAATATCTTCCCTTAAAAGCTACACAGA
AACATTC
Monomer type t22
TCAGAAATTGTTTGTCATGTGTGCTTTCAAATTACCAAGTTGAACCTACCTTGTG
ATTGAGCAGTTTTGAATCTCTCTTTTTGTGGAATCTGCAAGTGGATATTTTTAGCC
ATTTGCGGACTGTGGTGGAAAAGGAATTATCTTCAAATCCATTCTACACAGAAG
CAT
Monomer type t23
TCAGACAAACTTTTTGTGATGAGTGCATTGGTCACACAGAATTGAACCTCTCCTT
TGATTGAGCAATTCTGAAACACTCTTTCAGAGGGTCTGCAAGTGGATATTTTAG
AGCTTTGGGACAATTGTGGAAAAGTAAATATCTTCACATAGAAACTACACGGA
AGCATTC
Monomer type t24
TGAGAAACTTCTTTGGAGGTGTGCATTCAACTCACAGAGTTGAACCTATCTTTTC
ATTGAGCAGTTTTGAATCTCTCTTTTTGTAGACTCTGCTTGCAGATATTTGGAGA

GCTTTGAGGCCTATTGTGGAAAAGGAATCATCTTCACATAAAAACACACAGAA
GCACTC
Monomer type t25
TGAGAAACTTCTTTGTGAAGTGTGCATTCAACTCACAGAGTTGAACCTATCTTTT
GATTGAGAAGCTTTGAATCTCTCTTTTTGTAGAAGCTGCATGTGGATATTTGGAG
ACGTTTGTGGCCTATGGTAGAAAAGGCAATATCTTCAAATAAAAACTAGACAG
AAGCATTT
Monomer type t26
TGAGAAATTTCTCTGTGCTGTGTGCATTCATATCACATGGTTGAAACTACCTTTT
GGTTGAGCAGTTTTGAATCTCTCTTTTTGTAACATCTGCAATGGATATTTGGAGC
CCTTTGTGGTCTGTGGTGGAAAAGGAACTATCCTCAAATAAAAACTACACAGA
AGTATTC
Monomer type t27
CGAGAAACTTCCTTGTGATGTGTGCATTCATCTCACAGGGTTGAACCTTTGGTTT
GATTGAGCAGTTTTGAGACAATCTTTCCATAGAATCTGGAAGTGAATATTTGGA
GAACCTTGAGATCTATTTTGGAGAAGGAGATATCTTTATATGAAAACTGCACAG
AAGCATTC
Monomer type t28
TGAGAAACTTCTTTGTGATGTGTGCATTTATCTCACAGAGTTGAACCTTTGGTTT
GATTGAGCAGTTTTGAGATAATCTTTCCATAGAATCTGGAAGTGAATACTTGGA
TAACTTTGAGATCTATTTTGGAGAAGGAGATATCTTTATATAAAAACTGCACAG
AAGCATTC
Monomer type t29
TGAGAAACATCTTTGTGAGGTGTGCAATGAAGTCACAGAGTTGAAACTATGTTT
TGATTCAGCAGTTTTGAGTCTCTCTTTTTGCAGAATCTGCGAGTGGATATCTGGA
GAACTTGGAGGCCTATTTGGAAAAGGAAATATCTTCACATATAAACTATGCAGA
AGCATTT

Table S4. Canonical cascading 15mer consensus sequence.

Monomer type t1
TCAGAAACTTATTTGTGATGTGTGTCCTCAACTAACAGAGTTGAACCTTTGTTTT
GATACAGCAGTTTGGAAACACTCTTTTTGTAGAATCTACAAGTGGATATTTTGAG
AGCATTGAAAATTTCGTTGGAAGCGGAAAACCTTCATATAAAATCTAGACAG
AAGCATTC
Monomer type t2
TCAGAAACTGCTTTGTGATGTTTGCATTCAAGTCACCTAGTTGAACATTCCCTTT
CATAGAGCAGGTTTGAATCACTGTTTCTGTCGTATCTGGAAGTGGATATTTCGAG
CGTTTTCAGGCCTAAGGTGAGAAAGGAAATGTCTTCAAATAAGAACTAGACAG
AAGCATTC
Monomer type t3
TCAGAAACTTGTTTGTGACGTGTGTATTCAACTAACAGAGTTGAACCTTTCTTTTT
ACAGAGCAGCTTTGAAACCCTGTTTTTGTGGAATCTGCAATTGGAAATTTCGAT
AGTTCTGAGGATTTCGTTGGAAACGGGATTACAAATAGAAAGTAGACAGCAGC
ATTC
Monomer type t4
TCAGAAACTTCTTTGTAATGTTTGCATTCAACTCATAGAGTTGAACATTCCCTAT
CATACAGCAGGTTTGAAACACTCTTTTTGTAGTATGTGGAAGTGGACATTTGGA
GCGCTTTGAGGCCTACGGTGAAAAAGGAAATATCTTCCCATAAAAACTAGACA
GAAGCATTC
Monomer type t5
TCAGAATCTTCTTTGTGATGTATGCCCTCAATTCACAGAGTTGAACCTTTGTTTG
GATACAGCATTTTGGAAACATTCCTTTTGTAGAATCTGCAAGTTGATATTTGGAT
AGCTTTGAGGATTTCGTTGGAAACGGGAATATCTACATATAAAATCTAGACAGA
AGCATTC
Monomer type t6
TCAGAAACCTCTTTGTGATGTGTGTACTCAACTAACAGAGTTGAACCTTCCTTTT
CACAGAGCAGTTTGGAAACACTCTTTTTGTGGCATTTGCAAGTGGATATTTGGAT
AGCTTTGAGGATTTCGTTGGAAACGGGAATATTTTCATATAAAATCTAGACAGA
AGCATTC
Monomer type t7
TCAGAATCTTCTTTGTGATGTTTGCATTCAACTCATAGAGTTGAACATTCCCTTTC
ATACAGCACGTTTGAAACACACTTTGTGGAGTATGTGGAAATGGACATTTCGAG
CACTCTTAGGCCTAAGGTGAAAAGGGAAATATCTTCAAATAAAAACTAGTCAG
CAGCATTC
Monomer type t8
TCAGAACCTGCTTTGTGATGTTTGCATTCAACTCACAGAGCTGAACATTCCCGTT
CATAGAGCAGGTTTGAAACACTCTTTCTGTACTATCTGGAAGTGGACATTTCGA

GCGCTTTCAGGCCTATGGTGAAAAAGGAAACATCTTCAAATAAAAACTAGACA
GAAGCATTC
Monomer type t9
TCGCAATCTTGTTTGCCATGTGTGTACTCAACTAACAGAGTTGAACCTATCTTTT
GACAGAGCAGTTTTGAAACACTCTTTTTGTGGAATCTGCAAGTGGATATTTGGAT
AGCTTCGAGGATTTCGTTGGAAACGGGAATATCCTCATTTAAAATCTAGACGGA
AGCATTC

Table S5. Canonical Cascading 20mer consensus sequence.

Monomer type t1
TGAGAAACTTCTCTGTGAGGTGTGCTTTCAACTCACAGAGTTGAACCTATCTTTT
GATTGAGAAGTTTTGAATCTCTCTTTTTGTAGAAGCTGCATGTGGATATTTGGAG
ACGTTTGTGGCCTATGGTAGAAAAGGAAATATCTTCAAATAAAAACTAGACAG
ACGCATTT
Monomer type t2
TGAGAAACTTCTTTGGAGGTGTGCATTCAACTCACAGAGTTGAACCTATCTTTTC
ATTGAGCAGTTTTGAATCTCTCATTTTGTAGACTCTGCTCGCAGATATTTGGAGA
GCTTTGAGGCCTATTGTGGAAAAGGAAATATCTTCACATAAAAACACACAGAA
GCACTC
Monomer type t3
TCAGACAAACTTCTTTGTGATGAGTGCATTGGTCACACAGAATTGAACCTTCCCT
TTGATTGAGCAATTCTGAAACACTCTTTTGGAGGGTCTGCAAGTGGACATTTTAG
AGCTTTGGGACAACTGTGGAAAAGTAAATATCTTCACATAAAAACTACACGGA
AGCATTC
Monomer type t4
TCAGAAATTGTTTGTCATGTGTGCTTTCCAATTACCAAGTTGAACCTATCTTGTG
ATTGAGCAGTTTTGAATCTCTCTTTTTGTGGAATCGGCAAGTGGATATTTTTAGC
CCTTTGCGGACTGTGGTGGAAAAGGAATTATCTTCAAATCAATTCTACACAGAA
GCAT
Monomer type t5
TGAGAAACATCTTTGTGATGTGTGCATTCAGCTCACAGAGCTGGACCTAACTTTT
GAGTGACCAGTTTTGAATCTCTCTTTTTGTACAATATGCAAGTGGATATTTGGAG
CGATTTGAGGCCTACATTTGAAAATCAAATATCTTCCCTTAAAAACTACACAGA
AACATTC
Monomer type t6
TGAGAAACTTCTTTGTGATGTGTGCATTGATCTCACAGAGTTGAAAGTTTATTTT
GATTGAGCTGTTTTGAAACACTCTTTTTCTAGAATCTGCAAGTGGATAATTGGGG
AGATTTGAGGCATATTGTGGAAAAGCCAATATCTTCATATAAAAACTATACAGA
AACCTTC
Monomer type t7
TGAGAAACACCCTTGTGAGGTGTGCATTGAAGTCACAGAGTTAAACCTATCTTT
TGATTCAGCAGATTTGAATCTCTCTTTTTGCAGAATCTGCGAGTGGATATTTGGA
GTGCTTGGAAGCCTGCTGTGGAAAATCAAATATCTTCACAAAAAAAACTACAC
AGAAGCATTC
Monomer type t8
AGAGAAACTTCTCTGTGATGAGTGCATTCATCACACAGAGTTGAACATTTGTTT
AGATTTAGCAGTGTTGAGACAATCTTTCCGTAGAATCTTGAAGTGAATATTTGG

AGGGCTTTGAGACCTGCTTTGGAGAAGGAGATATCTTCATATAAAAACTACACA
GAAGCTTTC
Monomer type t9
TGTGAAACTTCTTTGCGATGTGTGCATTCAACTCACAGTGTTGAACCTATGTTTT
GATTGAGCAGTTTGGAATCTCTCTTTTTGTAGAATCTGCAAGTGAATATTTGGAG
CCCTATTTCGCCCTATACTGGAAAAGCAATTATCTTCAAATAAAAACTGCACAG
AAGCATTC
Monomer type t10
TGAGAAACTTCTTTGCGATGTTGGCATTCAACTCACAGAGTCGAATCTATCTTTT
GATAGAGCAGTTTTGTATCTCTCTTTTTGCAGAATCTGCAAGTGGATATTTGGAA
AGCTTTGAGGCCTATTGTGGAAAGGGAAATATCCTCAAATAAAAACTACCCAG
AAGCACTC
Monomer type t11
TGAAAAACATCTTTGGGATGTGTGCATTCAACTAACCGTGTTGAAACAATGTTTT
GATTGAGCAGCTTAGAATCTCTCTTTTTGTAGGAAATGCAAGTGGATATTTGGA
GCCCCATTTCGCCCTATGGTGGAAAACGAAACATACTCACAAAAAAGCTGCAG
AGAAGCATTC
Monomer type t12
TCCGAGAAACTTCTTTGTGATGTGTGCATTCAACTATCGGAGTTGAACCTATCTT
ATGATTGAGGAGTTTGGAAACACTCTTTGTAGAGTCTGCAAGTGGATATTTACA
GAGATTTGAGGCCTATTGTGGAAAAGGAAGTATCTTCACATAAAAACCACACA
GAAGCACTC
Monomer type t13
AGAGAAACTTCTTTGTGATGAATGCATTCATCACACAGAGTTGAACCTTTGTTTT
GATTTAGCAGTTTGAGACAATCTTTCCGTAGAATCTTGAAGTGAATATTTGGAG
GGCTTGGAGTTCTGTTTTAGAGAAGAAGATATCTTCATCAAAAACTACACAGAA
GCTT
Monomer type t14
TGAGAAACTACTTTGTGATGTGTGCATTCATCCCACAGAGTAGAACCTTTCTTTT
GATTGAGCAGTTTCGAAACACTCTTTTGGTGGAATCTGCAAGTGGACATTTGGA
AAGCTTTGAGGCCTATTGTGGAAAGGGAAATATCTTCAAATAAAAACCACCCA
GAAGTACTC
Monomer type t15
TGAGAAACTTCTCTGTCATACGTACATTCATCTCACAGGGTTGATCCTATTTCAT
GATTGAGCAGTTTTGGAACACTCTTTTTGTAGAATCTGCAAGTGAATATTTGGAG
CTCTTTGGGGCCTACTGTGGAAAAACAAATATCTTCACATAAAAACTACACAGA
AGCATTC
Monomer type t16
TGAGAAACTTTTTTTGTGATGTGGTCTTTCAGCTAATGGAGTAGAAACTATCTTT
TGATTGAGCAGTTTTGAATCTCTCTTTTTGCAGGATCTACGAGTGGATAATTGGA

GAACTTTGAGGCGTACTGTGGAAAGTCGAATATCTTCGCATAAAAACTACACAG
AAGCATTC
Monomer type t17
TGAGAAACATCCTTGTGAGGTGTGCACTGAAGTCACAGAGTTGAAACTGTCTTT
TGATTCAGCAGTTTTGAATCTCTCTTTTTGCAGAATCTGTGAGTGGATATTTGGA
GCGCTTTGAGGCCTACTGTGGAAAACCAAATATCTTCACATAAAAACTACACAG
AAGCATCC
Monomer type t18
TGAGAAACTTCTTTGTGATGTGGGCATTCATCTCACAGAGTTGAACCTTTGGTTT
GATTGAGCAGTTTTGAGACAATCTTTCCATAGAATCTGGAAGTGAATATTTGGA
GAACTTTGAGATCCATTTTGGAGAAGGAGATATCTTTATATAAAAACTACACAG
AAGCATTC
Monomer type t19
TGAGAAAATTCTCTGTGCTGTGTGCATTCATATCACATGGTTGAAACTACCTTTG
GATTGAGCAGTTTTGAATCTCACTTTTTGTACCATCTGCAATGGATATTTGGAGC
CCTTTCTGGTCTGTGGTGGAAAAGGAACTATCCTCAAATAGAAACTACACAGAA
GTACTC
Monomer type t20
TGAGAAACTTCTCTGTCATACGTACATTCATCTCACAGGGTTGATCCTATTTCAT
GATTGAGCAGTTTTGGAACACTCTTTTTGTAGAATCTGCAAGTGAATATTTGGGG
CCTACTGTGGAAAAACAAATATCTTCACATAAAAACTACACAGAAGCATTC
