# Supplementary figures and images for: Novel Concept of Alpha Satellite Cascading Higher-Order Repeats (HORs) and Precise Identification of 15mer and 20mer Cascading HORs in Complete T2T-CHM13 Assembly of Human Chromosome 15
Source: Int J Mol Sci. 2024 Apr 16;25(8):4395. doi: 10.3390/ijms25084395 (PMC11050224; doi:10.3390/ijms25084395)

Supplementary Fig. S2

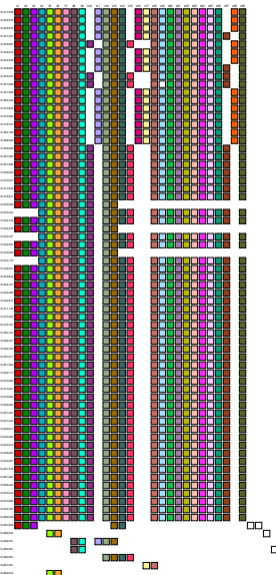

Supplement: Supplementary file 1 [file ijms-25-04395-s001.zip › Supplementary Fig S2.pdf]

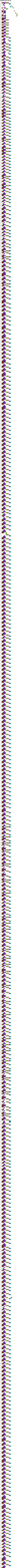

Supplement: Supplementary file 1 [file ijms-25-04395-s001.zip › Supplementary Fig S3.pdf]

Supplementary Fig. S4

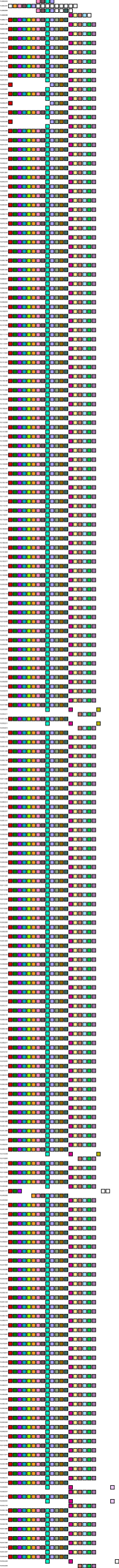

Supplement: Supplementary file 1 [file ijms-25-04395-s001.zip › Supplementary Fig S4.pdf]
